# Study of the probable genotoxic effects of Zolone (Phosalone) exposure in mice bone marrow derived cells

**DOI:** 10.1186/s41021-021-00191-5

**Published:** 2021-05-13

**Authors:** Zohre Khodabandeh, Mahmoud Etebari, Mehdi Aliomrani

**Affiliations:** 1 Department of Pharmacology and Toxicology, School of Pharmacy and Pharmaceutical Sciences, Isfahan University of Medical Sciences, Isfahan, Iran; 2 Department of Pharmacology and Toxicology, Isfahan Pharmaceutical Sciences Research Center, School of Pharmacy and Pharmaceutical Sciences, Isfahan University of Medical Sciences, Isfahan, Iran

**Keywords:** Organophosphate, Phosalone, Genotoxicity, Comet assay, Micronucleus test, Cyclophosphamide, Zolone

## Abstract

**Background and aim:**

Approximately, 2 million tonnes of pesticides are utilized annually worldwide. Phosalone (Pln), an organophosphorus pesticide, acts as an insecticide and acaricide to control pests of crops such as nuts, citrus fruits, pomegranates, stone fruits, grapes, potatoes, and artichokes. The purpose of this study was to evaluate the possible genotoxic effects following exposure to Pln in the cells derived from mouse red bone marrow.

**Materials and methods:**

Sixty mice were divided into 6 groups including cyclophosphamide (40 mg/kg, IP) and Pln (6, 12, 20, and 40 mg/kg) exposure by gavage. After 1 and 5 days of exposure, animals were euthanized and the genotoxicity assays were done on bone marrow extracted cells.

**Results:**

Comet assay shows a time and dose-dependent toxicity which further DNA degradation is observed after 5-day exposure (p < 0.05). Also, Pln significantly increased the MnPCE/PCE ratio after 12 and 20 mg/kg administration while no significant difference was reported between the doses of 6 and 40 mg/kg BW with the negative control group.

**Conclusion:**

Our results suggested a serious concern about its potential effects on biological life and related disease inductions. However further studies need to confirm the exact mechanism of Pln genotoxicity and the cause of diverse response of its activity at 40 mg/kg. This study also showed that increasing the dose of Pln reduces the MnNCE/Total cells ratio, which may indicate the possibility of bone marrow suppression. All of the above results emphasize the need to seriously limit the use of this compound as an agricultural pesticide.

## Introduction

Today, human beings are dealing with a wide range of diseases, including cancer, various autoimmune diseases such as lupus erythematosus, rheumatoid arthritis, multiple sclerosis, etc., which unfortunately, their prevalence rate is increasing day by day [1–4]. In general, the etiology of these diseases is still unknown. Although researchers initially considered the contribution of genetic factors to these diseases to be very high, recent research has shown that the development of these diseases is associated with other environmental factors such as exposure to toxic substances, infections, diet, and air pollution, which can play an even greater role than genetic factors [2, 5–7].

In general, one of the special applications of chemical compounds is their use as a pesticide in agriculture. Agricultural pesticides are substances in liquid, solid or gaseous forms applied to control the pests. Some of these pests, including insects, weeds and microbes, destroy the crops; hence, the use of agricultural pesticides is necessary to increase harvest efficiency and product quality[8]. Global pesticide consumption is estimated to reach more than 3.5 million tonnes by 2020. Meanwhile, 34 % of the world’s insecticide sales belong to the organophosphate insecticides. Significantly, the risks of chronic exposure to these compounds are due to their residue in food or due to poor working conditions, lack of personal protective equipment, and inadequate training on the hazards of construction and exposure to agricultural land, leading to immune system disorders, neurological diseases, endocrine disorders, miscarriage, degenerative diseases and cancers. As the glyphosate herbicide has been introduced for years, it has been found to be carcinogen and linked to many chronic diseases [9, 10].

Based on chemical structure, the pesticides are divided into four categories of organochlorine, organophosphorus, carbamates and pyrethroids. Organophosphorus pesticides are of the most widely used pesticide in agriculture, including Actellic, Dursban, Malathion, Diazinon and Phosalone (Pln) [11]. The Pln approved by the FAO and acts as an insecticide and acaricide to control pests of crops such as nuts, citrus fruits, pomegranates, stone fruits, grapes, potatoes and artichokes [12]. One of the most important mechanisms of toxicity following exposure to organophosphate agents is an increase in oxidative reactions in tissues due to a decrease in the effectiveness of enzymes involved in antioxidant defense, ultimately accelerating cell aging and possibly genetic damage of the cells. Interestingly, despite the widespread use of Pln, only its mutagenic effects on bacteria have been investigated by the Ames test so far [13].

Since Pln usage is a common and widely in the agricultural industry of developing countries, and no study has been performed to investigate the induced genotoxicity, the present study was conducted to investigate the association of acute exposure to the Pln and possible genotoxic effects in the cells derived from mouse bone marrow.

## Materials and methods

### Study animals

The current study was carried out on 60 Swiss albino male mice aged 6–8 weeks and weighing approximately 20–25 g, which were obtained from the animal house of faculty of Pharmacy at Isfahan University of Medical Sciences, Iran. The animals were kept in controlled conditions at a temperature of 20 ± 2 °C, humidity of about 50–60 %, 12–12 h light/dark cycle with easy and free access to water and food in accordance with the protocols of the National Committee for Research Ethics (IR.MUI.RESEARCH.REC.1397.405) [14, 15].

### Study design

The studied animals were divided into six groups of 10, including Group 1: receiving Pln-vehicle by gavage (negative control group); Group 2: receiving cyclophosphamide at a dose of 40 mg/kg body weight (BW) intraperitoneally (positive control); Groups 3 to 6: receiving Pln at doses of 6, 12, 20 and 40 mg/kg BW by gavage. The vehicle was prepared by dissolving 50 mg/ml of poloxamer powder in deionized water.

The genotoxicity tests were done 24 h after first exposure (1 day) and 24 h after five consecutive days’ exposure (5 days). Five mice were randomly selected and sampled at each time. It should be noted that the selection of time and implantation of these steps for the bone marrow were performed according to the latest OECD approved protocol.

### Bone marrow dissection

The animals were sacrificed at the end of study using an appropriate anesthesia agent (Ketamine 100 mg/kg and Xylazine 10 mg/kg, ip). Both femurs were immediately dissected and cleaned with a scalpel. After amputation of both heads of the bone, 2 cc of bone marrow was transferred into a microtube using fetal bovine serum (FBS). Cell pellets were obtained after centrifugation at 4000 rpm for 1 min to prepare smear on slides [16].

### Lymphocyte isolation

Ficoll solution was then used to isolate lymphocytes from whole blood. First, 1 ml of blood taken from each mouse was diluted by PBS at a ratio of 1: 1 in an EDTA-containing test tube. The diluted sample was poured slowly and without mixing onto a volume of Ficoll equal to the sample volume (2 ml) and centrifuged at 4^0^ C with 2800 rpm for 30 min. After centrifugation, four layers are formed, the upper layer containing plasma, the middle layer containing PBS and Ficoll, and the red blood cells in the bottom. The second layer, which contains a very small amount of lymphocytes, was carefully separated using a sampler and diluted with PBS at a ratio of 3: 1 then centrifuged at 1500 rpm for 5 min. Since the Ficoll solution is toxic this step was repeated two more times. Finally, the lymphocytes attached to the bottom of the Falcon tube were suspended in 1000 µl of PBS and 100 µl of which was used for a Comet assay.

### Comet assay

The alkaline comet assay was performed according to the previously described method with slight modification. Then, the lymphocytes isolated from the bone marrow were mixed with low melting agarose (0.5 %, Sigma-Aldrich, Germany) and were covered between two layer of normal melting agarose (1 %, Sigma-Aldrich, Germany). The slides were kept at room temperature and after the solidification, then the slides were immersed in cell wall lysis buffer containing 100nM Na2EDTA, 2.5 M NaCl, 10mM Triss buffer, 1 % Triton X-100 and 10 % DMSO at 4^0^ C in dark medium in order to denature the DNA strands and create a single strand. At the end of this phase slides were immersed in electrophoresis buffer containing 1 mM Na2EDTA, 300 mM NaOH with pH > 13 about 25 min at 25^0^ C. The electrophoresis was done at 300mA and 24 V for 45 min. After that the slides were neutralized with Triss buffer at pH 7.3 and stained with ethidium bromide (EtBr ,10 %) for 15 min. Finally, slides were analyzed by Olympus florescent microscope (Olympus, America, Melville, NY) and at least 50 cells from each sample were analyzed by Comet Score software to measure the parameters of Tail Length, DNA% in Tail and Tail Moment [17].

### Micronucleus test

Acridine orange staining solution with a final concentration of 1 mg/ml was first prepared by dissolving acridine orange powder in distilled water. Then, 10 µl of this solution was poured using a sampler on a corner of a completely clean glass slide, and then the solution was smeared onto the slide using a coverslip at a 45-degree angle. The slide was placed in a dark environment at room temperature to dry and used immediately. In the second step, 5 µl of the pellet containing the bone marrow isolated cells in the previous step was placed using a sampler in the center of the slide impregnated with completely dried acridine orange and immediately placed on the slide gently. Three slides were coded for each sample [16, 18].

The prepared slides were examined under the Olympus fluorescence microscope (Olympus, America, Melville, NY) using a blue filter (at a wavelength of 460nm). The images from the slides were routinely taken under the microscope at a magnification of 40X and a Z-shaped manner to avoid error and repetition. The normochromatic erythrocytes (NCEs: mature erythrocytes) were generally seen in green, and polychromatic erythrocytes (PCEs: immature erythrocytes) that had not yet lost their RNA or nucleus were observed in green with a yellow background and orange patches as opaque and asymmetric. Finally, the micronuclei (MN) within micronucleated normochromatic erythrocytes (MNNCEs) and micronucleated polychromatic erythrocytes (MNPCEs) were also visible between yellow and orange, which were completely spherical and smaller in size than the nucleus. The images were numbered and analyzed by Fijji image J software. To evaluate the frequency of micronuclei, the number of MNPCEs per 2000 PCEs per sample and 10,000 PCEs per each group were counted.

### Statistical analysis

The results of all groups were presented as Mean ± SD. Graphpad Prism version 8 Software was used for statistical analysis. Statistical comparison of groups was performed using one-way ANOVA with Tukey’s post-hoc test. P-value < 0.05 was statistically considered as significance level.

## Results

### Weight and behavioral changes

Weight and behavioral changes in both of the experimental and control groups were measured and recorded at the beginning of the delivery time (baseline weight) and also on the day of sampling. The results showed that there were no significant changes between groups (Data were not shown).

### Comet assay

To evaluate the genotoxicity of Pln, the extent of DNA damage in lymphocytes of all groups tested by the Comet assay was compared at 24 h after the first dose of Pln gavage and after 5 consecutive days. Three factors of tail length, DNA% in tail and tail moment were used to measure and compare the degrees of destruction. The following diagrams show the results of the Comet assay in different groups.

**Fig. 1:**
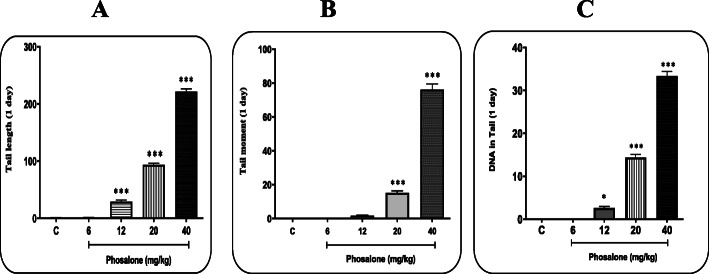
Comet assay results in mice following 1 day of Phosalone administration at the doses of 6, 12, 20 and 40 mg/kg BW for three parameters of (**a**) Tail length, (**b**) Tail moment and (**c**) DNA% in tail; C: negative control group (receiving Pln-carrier complex). Results are reported as Mean ± SEM; * and ***: significant difference in comparison with negative control group (p < 0.05 and p < 0.001, respectively)

**Fig. 2:**
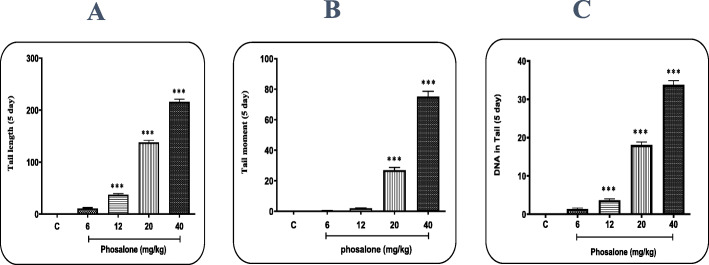
Comet assay results in mice following 5 days of Phosalone administration at the doses of 6, 12, 20 and 40 mg/kg BW for three parameters of (**a**) Tail length, (**b**) Tail moment and (**c**) DNA% in tail; Nctrl: negative control group (receiving Pln-carrier complex). Results are reported as Mean ± SEM; ***: significant difference with negative control group (p < 0.001)

The tail length in the groups receiving Pln for 1 day (Fig. 1 a) and 5 days (Fig. 2 a) at the doses of 12, 20 and 40 mg/kg BW was significantly different from the negative control group (p < 0.05). The time factor is also effective in increasing this degradation so that further DNA degradation is observed in 5-day groups than in 1-day groups.

The tail moment was very low in the negative control group. The results showed that receiving Pln at the doses of 20 and 40 mg/kg BW for 1 day compared to the negative control group significantly (p < 0.05) caused an increase in the tail moment (Fig. 1b). Moreover, an increase in the tail moment following 5 days of Pln administration was reported at all doses compared to the negative control group (Fig. 2b). Also there was a significant difference in terms of changes in DNA% in tail between the groups receiving Pln at all doses except 6 mg/kg and the negative control (Figs. 1 and 2 c).

### Micronucleus test

### The Pln administration for 1 day

**Fig. 3:**
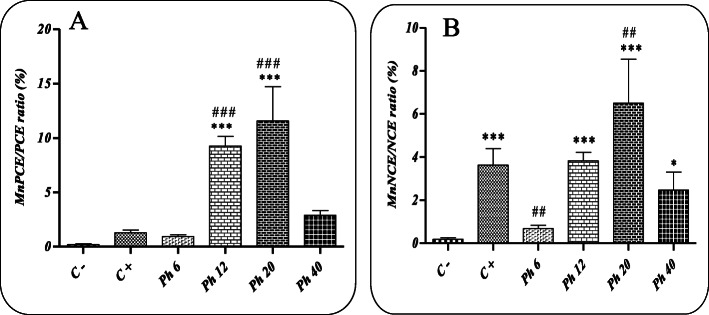
Results of micronucleus evaluation and the effect of different concentrations of Phosalone administration for 1 day at the doses of 6, 12, 20 and 40 mg/kg BW; (A): MnPCE/PCE ratio (%); (B): MnNCE/NCE ratio (%); (C^−^): negative control group (receiving Pln-carrier complex); (C^+^): Cyclophosphamide receiving group (40 mg/kg); * and ***: a significant difference in comparison with the negative control group (p < 0.05, p < 0.001, respectively). ^##^ and ^###^ indicates a significant difference in comparison with the positive control group (p < 0.01, p < 0.001, respectively)

In Fig. 3, where the MnPCE/PCE ratio (%) is reported, the frequency of micronuclei is very low in the negative control group receiving only Pln-carrier complex. The results showed that the administration of Pln at the doses of 12 and 20 mg/kg BW for 1 day compared to the negative control group significantly (p < 0.05) increased the MnPCE/PCE ratio, while no significant difference was reported between the doses of 6 and 40 mg/kg BW with the negative control group.

The administration of Pln for 1 day at all doses except 6 mg/kg BW was able to significantly (p < 0.05) increase the MnNCE/NCE ratio (%) compared to the negative control group receiving only Pln-carrier complex.

### The Pln administration for 5 days

**Fig. 4:**
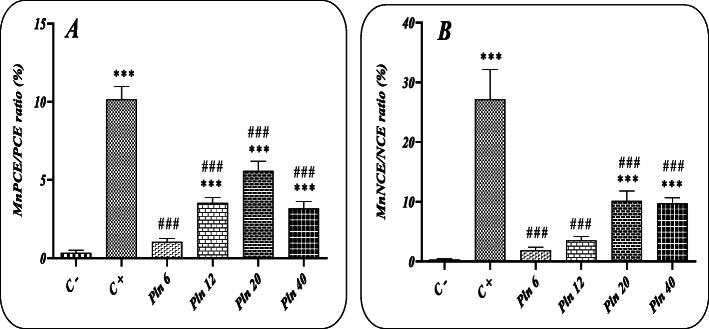
Results of micronucleus evaluation and the effect of different concentrations of Pln received for 5 day at the doses of 6, 12, 20 and 40 mg/kg BW; (A): MnPCE/PCE ratio (%); (B): MnNCE/NCE ratio (%); (C^−^): negative control group (receiving Pln-carrier complex); (C^+^): Cyclophosphamide receiving group (40 mg/kg); ***: a significant difference with the negative control group (p < 0.001). ^###^ indicate a significant difference in comparison with the positive control group (p < 0.001)

There was a significant difference in the MnPCE/PCE ratio after receiving 5 days of Pln at the doses of 12, 20 and 40 mg/kg BW compared with the control group, so that the doses of 12, 20 and 40 mg/kg BW of Pln caused a significant increase in the MnPCE/PCE ratio. The effect of time is also visible in the extent of damage to the genetic material (Fig. 4 a). Our results showed that the Pln administration after 5 days at the doses of 12, 20 and 40 mg/kg BW caused an increase in the MnNCE/NCE ratio compared to the negative control group, but this effect was not reported at the lowest Pln dose.

### MnPCE/Total cells ratio after Pln administration for 1 and 5 days

**Fig. 5:**
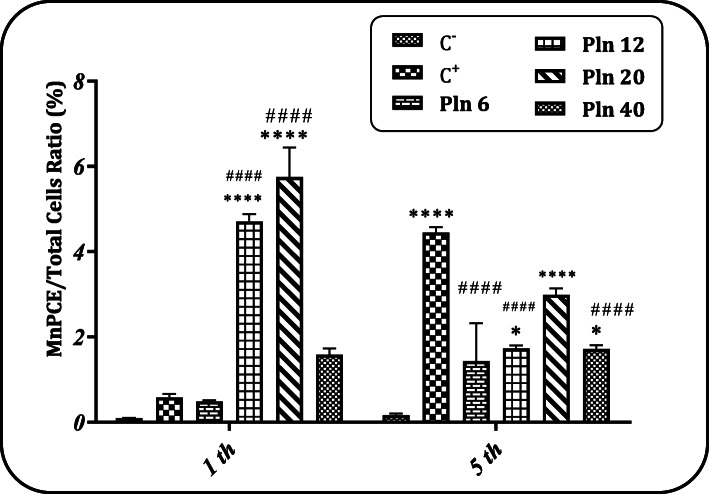
MnPCE/Total cells ratio (%). Evaluation of the effect of different doses of Pln received for 1 and 5 days (6, 12, 20 and 40 mg/kg BW) on the MnPCE/PCE ratio (%); (C^−^): a negative control group (receiving Pln-carrier complex); (C^+^): Cyclophosphamide receiving group (40 mg/kg); * and ****: a significant difference with the negative control group (p < 0.05, p < 0.0001, respectively). ^####^ indicate a significant difference in comparison with the positive control group (p < 0.0001)

As shown in Fig. 5, the administration of Pln for 1 day at the doses of 12 and 20 mg/kg BW was able to significantly (p < 0.05) increase the MnPCE/Total cells ratio (%) compared to the negative control group. However, there was a significant difference (p < 0.05) in MnPCE/Total cells ratio between all doses of Pln except 6 mg/kg BW and the negative control group after 5 days of administration.

### MnNCE/Total cells ratio after Pln administration for 1 and 5 days

**Fig. 6:**
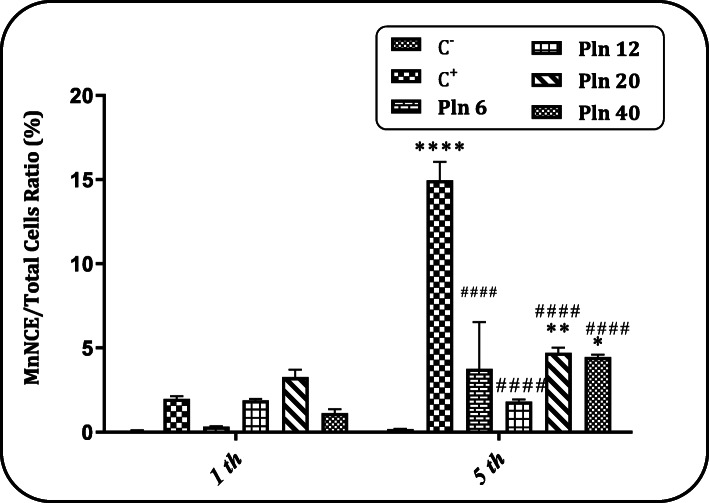
MnNCE/Total cells ratio (%). Evaluation of the effect of different doses of Pln received for 1 and 5 days (6, 12, 20 and 40 mg/kg BW) on the MnPCE/PCE ratio (%); (C^−^): a negative control group (receiving Pln-carrier complex); (C^+^): Cyclophosphamide receiving group (40 mg/kg); *: a significant difference with the negative control group (p < 0.05). ^####^ indicate a significant difference in comparison with the positive control group (p < 0.0001)

**Fig. 7:**
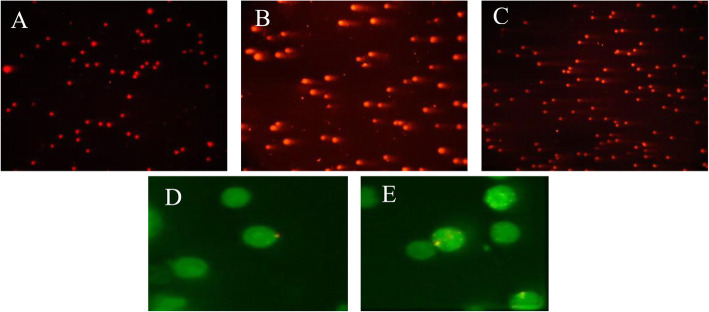
Pictures taken for Comet assay; (**a**) negative control, (**b**) positive control (**c**) cells treated with 20 mg/kg of Phosalone. Pictures taken for micronucleus test; MNNCE (**d**) and MNPCE (**e**)

Figure 6 shows the MnNCE/Total cells ratio after receiving Pln for 1 and 5 days. In administration for 1 day, the results showed that there was no significant difference in the MnNCE/Total cells ratio between any of the groups receiving Pln at all doses and negative control. In administration for 5 days, the results showed that there was a significant difference (p < 0.05) in the MnNCE/Total cells ratio between the highest doses of Pln and the negative control group. The sample pictures were taken from comet assay and micronucleus test are presented in Fig. 7 (a-e).

## Discussion

Approximately, 2 million tonnes of pesticides are utilized annually worldwide [19]. The accumulation of these substances in the environment made a serious threat to human being through the food chain and drinking water. More than 100 organophosphorus compounds are being used in which the majority of them are mutagenic or either linked to the further cancers developments. In which oxidative stress induction and DNA alkylation are the most proved molecular mechanism of pesticides genotoxicity [20, 21].

Phosalone or S-6-chloro-2,3-dihydro-2-oxobenzoxazol-3-ylmethyl O,O-diethyl phosphorodithioate is stable (< 10 % degredation after 4 weeks) in water at pH 5–7 with a half-lifes of 9 days at pH 9 [22]. According to the WHO, the Pln was classified as a moderately dangerous pesticide (Class II) and banned in European countries, but it is still used as one of the most common insecticides in fruit and other agricultural products in many developing and underdeveloped countries in the field of agriculture. This pesticide is also known as one of the common insecticides used to control the Colorado potato beetle[23]. Several studies were measured the Pln residue concentration in fruits including tomato (0.005 mg/kg) [24, 25], pistachios [26] and so on. The hypothetical pathway for metabolism in mice is probably the hydrolysis of Pln to O, O-diethylphosphorodithioate and mercapto-3-thiomethyl 6-chlorobenzoxazolone. It is subsequently converted to sulfide, which is oxidized to sulfoxide and sulfone. Tissues such as the brain, liver, fat, and skeletal muscle contained high levels of sulfide has higher Pln concentration. The Pln has been reported to be extensively excreted mainly through urine after metabolization [27].

The acute toxicity of Pln has been studied earlier in female rats with an oral LD50 about 110 mg/kg while the the acute percutaneous LD50 of it was around 1500 mg/kg [12]. Up to days, little information about the genotoxicity of Pln is available. However, studies about the effects of Pln and its oxidative stress induction have indicated that it has the potential of genotoxicity induction. The current study investigated the possible genotoxic effects following exposure to Pln as an organophosphate pesticide in cells derived from mouse red bone marrow, and the results of which revealed that the DNA damage was higher in the lymphocytes isolated from groups receiving higher doses of Pln. It is noteworthy that the time factor was also effective in increasing this degradation (DNA damage in 5 days was far more than in 1 day). These results are consistent with previous studies, which demonstrated that the exposure to pesticides causes DNA damage.

In this study statistical significant differences between groups weight changes and behavioral effects were not seen. However, previous studies have been shown that Pln exposure significantly reduce total body weight gain in rats [28], which may be related to the Pln effect on the central structures involved in controlling food intake in the hypothalamus. It could be inferred that this effect is related to the duration and dose of exposure.

Like other members of the organophosphate pesticide family, the Pln can increase the level of ROS in human tissues, which is associated with a decrease in the activity level of antioxidant enzymes. High levels of ROS cause membrane damage and cell function imbalance through increased LPO [29, 30]. Faster cell aging and the development of DNA and RNA changes are the ultimate consequences of increased ROS, eventually leading to cancer and gene mutations. Comet assay shows an increase in all parameters examined in the extent of DNA damage with increasing Pln dose. DNA percent in tail, tail length and tail moment of the groups that received the Pln, vehicle and cyclophosphamide had a significant difference as compared with the negative control group except the group receiving 6 mg/kg BW. In accordance with our results high DNA fragmentation, RNA damage, decreased total antioxidant capacity, and glutathione peroxidase have also been reported in the testicular tissue of Pln-receiving rats [31]. Pln has been shown to be associated with aging in MEFs through an increase in oxidative stress factors, ROS production, expression of aging-related genes, inflammatory cytokines, cell cycle arrest, apoptosis and necrosis [31]. A study investigated the effects of chronic exposure to Pln and histological changes in the common carp. It was suggested that the Pln concentrations are related to the lipid peroxidation and antioxidant defense system defects because of elevating the ROS formation and caused the complications in the fish [32].

The micronucleus test has been suggested as a sensitive test to evaluate cumulative effects of genotoxic substances exposure [33]. However, the results need more confirmation due to various clastogenic mechanisms of toxic substances. Detected micronuclei in the cytoplasm of the cells mainly compromised large chromosomal fragments or the whole chromosome which has been left behind during cellular division [34]. As shown the frequency of micronuclei in the negative control group receiving only the Pln-carrier complex was very low, indicating the non-cytotoxic nature of the selective carrier. The results demonstrated that the administration of Pln at the doses of 12 and 20 mg/kg BW significantly increased the MnPCE/PCE ratio compared to the negative control group, while there was no significant difference between the doses of 6 and 40 mg/kg BW with the negative control group. A sharp decrease in the MnPCE/PCE ratio at the dose of 40 mg/kg BW is very interesting and may be due to the involvement of the body’s defense systems and the spleen to clear the toxic substances. The time effect is also evident in the extent of damage to the cellular genetic material as observed that this ratio significantly increased after 5 days’ exposure in all doses except 6 mg/kg.

Our results indicated that Pln increased clastogenic damage after acute exposure even more than cyclophosphamide in the MN test, but after chronic exposure, the frequency of micronuclei in PCE cells were decreased in comparison to the cyclophosphamide treated group. Also, Pln showed its potential cytotoxic effects by decreasing the total cells number (PCE and NCE cells) time dependently. In accordance with our results Ali and his colleagues has been reported that chlorpyrifos exposure MN induction increased concentration dependently and decreased time dependently [35].

As seen in the results, the administration of Pln for 1 day at the doses of 12 and 20 mg/kg BW could significantly increase (p < 0.05) the MnPCE/Total cells ratio (%) compared to the negative control group. And after 5 days, the results revealed a significant difference in the MnPCE/Total cells ratio between all concentrations of Pln except 6 mg/kg BW and the negative control group. Contrary to the observations of the results of 1-day administration, there was a significant difference between the positive control group and the negative control group after 5 days of administration. In addition, our results documented a significant difference (p < 0.05) between the groups receiving 6, 12 and 40 mg/kg BW doses for 5 days and the positive control group.

Detected damages measured with comet assay are mainly strand breaks which probably occurred after action of repair systems. With this regards increased comet score depended to the time and concentration of the Pln exposure explained. However, in micronucleus test we observed that MnNCE count increased after 5 days which may be the result of MnPCEs maturation. But the MnPCEs are decreased after 5 days in which it could be explain that total number of PCE cells were decreased due to the cytotoxic effects of Pln. Put on the whole, the Pln disturb DNA integrity, but the major mechanism(s) of its interaction is unknown.

The pesticides may cause oxidative stress in multiple ways, thereby disrupting the balance between ROS and antioxidant mechanisms [36]. This imbalance can damage cellular components including proteins, lipids, DNA and RNA. The ROS can be involved in a variety of DNA damage, including double-strand breakage, as well as the oxidation of bases that can be easily detected by the comet assay [37]. The Pln has been shown to increase ROS levels in human tissues, increase LPO levels, and decrease the activity of antioxidant enzymes in erythrocytes in vitro by increasing ROS. Also the Pln has been reported to cause acute pancreatic toxicity [38]. In addition to inhibiting acetylcholinesterase by increasing ROS, it causes destructive effects on various tissues [39].

## Conclusion

The results obtained from the present study demonstrated the dose-dependent and time-dependent genotoxic effects of Pln. The administration of Pln at the doses of 12, 20 and 40 mg/kg BW increased the cell genetic damage compared with the negative control group. In addition, the Pln by increasing the exposure time of evolving red blood cells causes more damage to their genetic material, which may be repaired during the conversion of PCE to NCE. This study also showed that increasing the dose of Pln reduces the MnNCE/Total cells ratio, which may indicate the possibility of bone marrow suppression. Our results on the genotoxic potential of Pln suggested a serious concern about its potential effects on biological life and related disease inductions. However further studies need to confirm the exact mechanism of Pln genotoxicity and the cause of diverse response of its activity at 40 mg/kg. All of the above results emphasize the need to seriously limit the use of this compound as an agricultural pesticide.

from Isfahan Pharmaceutical Science Research Center, Isfahan University of Medical Sciences, Isfahan, Iran.

## Data Availability

The datasets used and/or analyzed during the current study are available from the corresponding author on reasonable request.

## Abbreviations

Pln: Phosalone
FAO: Food and Agriculture Organization
BW: Body weight
OECD: Organization for Economic Co-operation and Development
FBS: Fetal bovine serum
ml: milliliter
ip: Interaperitoneal
EDTA: Ethylenediaminetetraacetic acid
DMSO: DMSO: Dimethyl sulfoxide
DNA: Deoxyribonucleic acid
mM: Millimolar
NCEs: Normochromatic erythrocytes
PCEs: Polychromatic erythrocytes
MNNCEs: Micronucleated normochromatic erythrocytes
MNPCEs: Micronucleated polychromatic erythrocytes
WHO: World Health Organization
LD50: Lethal dose
ROS: Reactive oxygen species
